# Rapidly progressive psychotic symptoms triggered by infection in a patient with methylenetetrahydrofolate reductase deficiency: a case report

**DOI:** 10.1186/s12883-017-0827-0

**Published:** 2017-02-28

**Authors:** Shin Iida, Masataka Nakamura, Shinya Asayama, Takenobu Kunieda, Satoshi Kaneko, Hitoshi Osaka, Hirofumi Kusaka

**Affiliations:** 1 Department of Neurology, Kansai Medical University, 2-5-1, Shinmachi, Hirakata, Osaka 5731010 Japan; 2 Department of Pediatrics, Jichi Medical School, 3311-1, Yakushiji, Shimotsuke-shi, Tochigi 3290498 Japan

**Keywords:** MTHFR, Betaine, Spastic paraplegia, Psychosis, Leukoencephalopathy

## Abstract

**Background:**

Methylenetetrahydrofolate reductase (MTHFR) deficiency is a rare inborn error of metabolism inherited in autosomal recessive pattern and is associated with a wide spectrum of neurological abnormalities.

**Case presentation:**

We herein describe a 15-year-old boy with MTHFR deficiency who presented with a slowly progressive decline of school performance and a spastic gait. Rapidly deteriorating psychosis and repetitive seizures triggered by a febrile infection prompted neurological investigation. He had significantly elevated total plasma homocysteine and urinary homocystine levels, as well as a decreased plasma methionine level. Brain magnetic resonance imaging (MRI) revealed leukoencephalopathy. DNA gene sequencing showed c.446_447 del GC ins TT and c.137G > A, and c.665C > T heterozygous mutations in the MTHFR gene of the patient. Oral administration of betaine drastically improved his clinical symptoms within a few months. After 8 months of treatment, his total plasma homocysteine level moderately decreased; and the plasma methionine concentration became normalized. Furthermore, the white matter lesions on MRI had disappeared.

**Conclusion:**

This patient demonstrates the possibility that MTHFR deficiency should be considered in mentally retarded adolescents who display an abnormally elevated plasma level of homocysteine in association with progressive neurological dysfunction and leukoencephalopathy. Febrile infections may be an aggravating factor in patients with MTHFR deficiency.

## Background

**Fig. 1:**
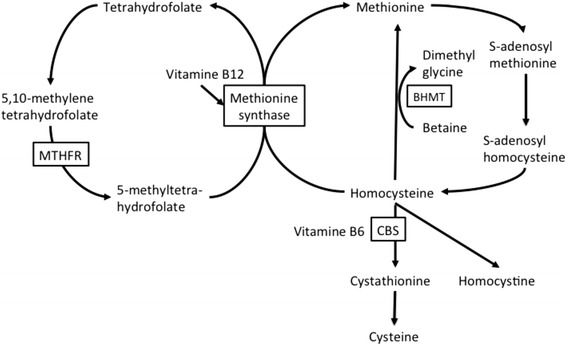
An overview of methionine and homocysteine metabolism. MTHFR, Methylenetetrahydrofolate reductase; CBS, cystathionine beta-synthase; BHMT, betaine-homocysteine S-methyltransferase

5,10-Methylenetetrahydrofolate reductase (MTHFR) deficiency is a rare autosomal recessive disease. The enzyme MTHFR catalyzes the conversion of 5,10-methylenetetrahydrofolate to 5-methyltetrahydrofolate, which serves as a methyl donor in the remethylation of homocysteine to methionine (Fig. 1). Therefore, patients with this deficiency show hyperhomocysteinemia, hypomethioninaemia and usually low folate levels. A broad spectrum of clinical symptoms can appear neonatally, in early or late childhood, or even in the adult. The infantile presentation is the most clinically severe and includes hypotonia, seizures, apneas and/or coma. Adolescents or adults may present with mental retardation, motor and gait disturbance, seizures, psychiatric manifestations, and thrombosis [1]. We herein report the case of a 15-year-old patient with MTHFR deficiency presenting with a slowly progressive mental decline and spastic paraplegia, followed by rapidly progressive psychosis and repetitive seizures triggered by infection that successfully responded to betaine treatment.

## Case presentation

A 15-year-old Japanese boy was referred to our hospital for rapidly progressive psychiatric and neurological symptoms. His growth and development had been normal until the age of 11, when he began to experience academic difficulties.

At the age of 13 years, he visited a local hospital because of a 6-month history of slowly progressive difficulty in walking. Neurological examination revealed a spastic gait and marked lower limb hyper-reflexia. However, brain and spinal magnetic resonance imaging (MRI) showed no abnormalities. His total plasma homocysteine level was 216 nmol/ml (normal, 3.7-12.5 nmol/ml); and serum folic acid and cobalamine levels were 1.7 ng/ml (normal, 3.6–12.9 ng/ml) and 397 pg/ml (normal, 233–914 pg/ml), respectively. The plasma concentrations of very-long-chain fatty acids were all within normal limits. Based on a tentative diagnosis of subacute combined degeneration of the spinal cord secondary to folic acid deficiency, folic acid (15 mg/day) therapy was started. However, his gait disturbance gradually worsened.

**Fig. 2:**
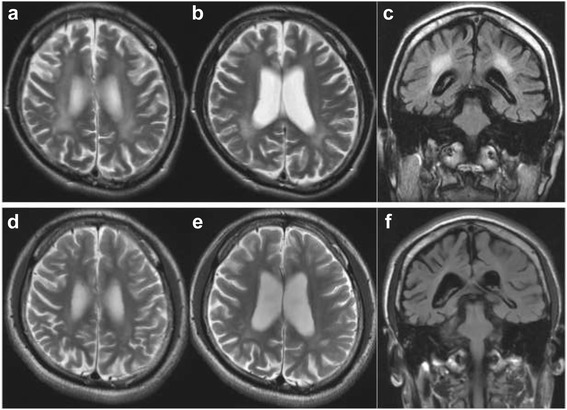
**a**-**c** Axial and coronal T2 weighted magnetic resonance imaging (MRI) images and axial fluid-attenuated inversion recovery (FLAIR) MRI image obtained at age 15 reveal a high-intensity area in the white matter around the posterior horns of the lateral ventricles. **d**-**f** Axial and coronal T2 weighted MRI images and FLAIR MRI image obtained at age 16 (8 months after betaine treatment) show resolution of the hyper-intensity in the white matter

At the age of 15 years, he began to show deficits in motivation and effort. One week later, he was immediately admitted to a hospital because of a high fever and acute diarrhea with vomiting caused by viral gastroenteritis that had started the previous evening. After admission, he rapidly developed psychotic symptoms, particularly delusions and auditory hallucinations. His general condition was improved by rehydration, and he was discharged 3 days later with a diagnosis of hospital-induced delirium. However, his psychotic symptoms, such as depression, delusions, hallucinations, and anorexia, worsened over the next 2 weeks. Furthermore, he experienced several episodes of generalized convulsions, for which levetiracetam was prescribed (1000 mg/day). He was repeatedly in and out of another local hospital because of dehydration due to a refusal to eat caused by his psychotic state or convulsions. He was treated with risperidone for a month, but still suffered from delusions, hallucination, and failure to eat. A brain MRI showed generalized atrophy and hyperintensity in the white matter around the posterior horns of the lateral ventricles on T2-weighted and fluid-attenuated inversion recovery (FLAIR) images (Fig. 2a-c). An electroencephalogram showed a diffusely low-amplitude background activity without epileptic discharge. The results of a nerve conduction study and biochemical examination including lysosomal enzymatic activities were both normal. He became severely apathetic and bedridden within two months after infectious gastroenteritis. Therefore, he was transferred to our hospital for further investigation.

On admission, no abnormal facial appearance or skeletal anomalies were noted. Neurological examination revealed a fluctuating level of consciousness with confusion, delusion, and abnormal behavior. Cranial nerves were normal. Marked spasticity of the lower limbs was noticed, with hyper-reflexia in both arms and legs. There was also sustained clonus at the ankles following sudden passive dorsiflexion of the foot. However, pathological reflexes (Babinski and Chaddock signs) were negative. Serum folic acid and cobalamine levels were elevated (more than 20 ng/ml and 1120 pg/ml, respectively). Plasma amino acid analysis showed an elevated level of total homocysteine (229.9 nmol/ml) and a decreased methionine level (10.9 nmol/ml; normal, 19.2-32.7 nmol/ml). Cerebrospinal fluid analysis indicated normal values. Gas chromatographic-mass spectrometric urinary metabolome analysis revealed a markedly elevated urinary homocystine level of 19.7 mmol/mol total creatinine (normal, not detected) and undetectable urinary methionine levels (−3.0 standard deviation; SD). His urinary methylmalonic acid level was normal (−0.3 SD). These results of the amino acid analyses were highly suggestive of MTHFR deficiency. Therefore, MTHFR gene sequencing was undertaken; and the results showed compound heterozygous mutations c.446_447 del GC ins TT (p.Gly149Val) and c.137G > A (p.Arg46Gln). In addition, heterozygous c. 665C > T (p.Ala222Val) polymorphism was noted. Multivitamins including vitamins B1, B6, and B12 (750 μg/day) as well as folic acid were administered. Subsequently, oral substitution treatment with betaine was initiated at a dosage of 3 g/day, which was slowly increased to 9 g/day. Over a few months, consciousness, delusion, and abnormal behavior dramatically improved, and he became able to sit in a wheelchair. After 8 months of therapy, the earlier white matter hyperintensities on T2-weighted and FLAIR images had resolved (Fig. 2d-f). Moreover, his total plasma homocysteine level had decreased to around half of its initial value (91.2 nmol/ml); and the plasma methionine level had normalized (20.4 nmol/ml). Thereafter, low specific activity of MTHFR was observed in skin fibroblasts cultured from the patient (2.8 nmol/h/mg protein; normal control values in 75 normal subjects, 25.3 ± 8.9 nmol/h/mg protein). MTHFR gene sequencing of his mother revealed mutation c.137G > A and polymorphism c. 665C > T, both in the heterozygous state. Following 2 years of clinical follow-up, the patient had no psychiatric symptoms (apathy, delusion or hallucination) or relapse, and he became able to stand up and walk with the aid of a walker.

## Discussion

Our patient had two compound missense mutations, c.446_447 del GC ins TT and c.137G > A, in the heterozygous state in addition to a c. 665C > T functional polymorphism. The tandem missense mutation c.446_447 del GC ins TT had already been described in Japanese patients with MTHFR deficiency [2–4]. These patients had a homozygous or heterozygous mutation and displayed decreased MTHFR activity. The MTHFR gene polymorphism c. 665C > T is also known to be associated with reduced enzyme activity [5]. Genetic analysis of the present patient’s mother revealed the presence of c.137G > A and c. 665C > T, indicating that pathogenicity of our patient could have been due to the combined effect of tandem missense mutation c.446_447 del GC ins TT and common polymorphism c. 665C > T.

Newborn screening for homocystinuria is based on the identification of an increased level of plasma methionine. MTHFR deficiency causes a low methionine level and is therefore not detected by current screening. Unless clinicians maintain a high index of suspicion and are aware of varied clinical presentations, this diagnosis is often missed. The clinical presentation and age at onset of MTHFR deficiency varies greatly depending on the degree of enzyme deficiency [6]. Patients with severe MTHFR deficiency (0-20% residual enzyme activity) in infancy or adolescence present with developmental delay, severe mental retardation, motor and gait dysfunction, seizures, psychiatric disturbances, and other neurological abnormalities. Our patient displayed decreased MTHFR activity (11% of control activity) and had a clinical pattern and age at presentation consistent with a severe deficiency.

Previously, rapidly deteriorating psychosis was reported in the case of adolesecent- or adult-onset MTHFR deficiency [7–10]. The reason for rapid deterioration in these patients is not known; but the lack of dietary supplements, the use of nitrous oxide during general anesthesia, and long-term use of the antiepileptic drug phenytoin have been reported as risk factors for acute neurological deterioration [3, 11]. So, we speculate that the change in nutritional status through reductions in dietary intake and intestinal absorption due to gastroenteritis might have been responsible for the rapid deterioration seen in our patient. Moreover, prolonged undernutrition due to refusal to eat could have aggravated his psychiatric symptoms. Another possible cause is that the common MTHFR gene polymorphism (c. 665C > T) increases the thermolability of the enzyme [5]. Thus, a high fever may potentially worsen psychiatric symptoms by causing inactivation of the enzyme activity. Therefore, febrile infections may be an aggravating factor in patients with MTHFR deficiency.

The biochemical therapeutic goals in MTHFR deficiency are aimed at reducing plasma homocysteine and normalizing methionine levels. Betaine is an endogenous catabolite of choline, and high doses of betaine are the mainstay of therapy for patients with MTHFR deficiency. The beneficial effect of betaine is mediated through betaine-homocysteine methyltransferase with the use of an alternate methyl donor for remethylation of homocysteine to methionine (Fig. 1) [12]. Indeed, betaine supplementation has been shown to decrease homocysteine levels and normalize methionine levels in patients with MTHFR deficiency [12]. Folic acid and supplementation with vitamins B6 and B12 also play key roles in converting homocysteine into methionine. As expected in our case, the supplementation with betaine and multi-vitamins resulted in a moderate decrease in the level of total plasma homocysteine and normalization of plasma methionine, that were associated with the disappearance of psychosis and leukoencephalopathy and the recovery of motor functions.

## Conclusions

We reported the case of a school-aged boy with severe MTHFR deficiency who presented with a slowly progressive mental decline and spastic paraplegia and who later developed rapidly progressive psychosis and repetitive seizures following infectious gastroenteritis. Patients with MTHFR deficiency are sensitive to febrile infections, which may trigger new-onset neurological or psychic symptoms, or worsen existing symptoms. Since MTHFR deficiency is one of the treatable metabolic disorders not identified by current newborn mass screening, we should consider the possibility of MTHFR deficiency in adolescents with mental retardation who display abnormally elevated homocysteine in association with progressive neurological or psychiatric dysfunction and leukoencephalopathy.

## Abbreviations

FLAIR: Fluid-attenuated inversion recovery
MRI: Magnetic resonance imaging
MTHFR: Methylenetetrahydrofolate reductase
SD: Standard deviation
